# Editorial: Rapid and Cost-Effective Technologies to Detect the Pathogens in Food and Environment

**DOI:** 10.3389/fmicb.2022.834774

**Published:** 2022-06-20

**Authors:** Xuejun Ma, Yi-Wei Tang, Xiyang Wu, Joseph O. Falkinham

**Affiliations:** ^1^Chinese Center for Disease Control and Prevention, Beijing, China; ^2^Cepheid, Sunnyvale, CA, United States; ^3^Jinan University, Guangzhou, China; ^4^Virginia Tech, Blacksburg, VA, United States

**Keywords:** microbial detection, plants, food, wastewater pathogens, DNA-based detection, immunomagnetic, CRISPR

The value of this Research Topic lies in the breadth of microorganisms as subjects of interests, the applications of the technologies, and the different approaches. That breadth serves to guide anyone with interests in pathogen detection; all serve as instructive examples. The articles fall into two categories: (1) methods for detection of specific food-borne pathogens and (2) methods for detection of different food-borne pathogens, and (3) methods to overcome the challenges of different starting materials.

A wide variety of organisms are described, including: *Listeria* (Zhang X. et al.)*, Aspergillus* (Martinez et al.)*, Salmonella* (An et al.; Liu et al.)*, Phytopythium* (Wang et al.)*, Escherichia* (Nakae et al.; Yang et al.), *Vibrio* (Xiao et al.; Zhang X. et al.), and Norovirus (Zuo et al.). Each provides its own challenges to detection method development and therefore serve as guides to the challenges of detection in different starting materials. The breadth of methods for detection of foodborne pathogens is likewise quite wide. Metagenomics (Zhang L. et al.), CRISPR (Xiao et al.), Lab-on-Chip (Yang et al.), immunomagnetic enrichment (Gao et al.), bacteriophage-linked detection (Wisuthiphaet et al.), and other amplification methods (Zuo et al.; Wang et al.) are included to provide guidance in the choice of experimental approaches. Finally, the volume even has a contribution for detection of microbial DNA, applicable to a wide range of starting samples (e.g., water, wastewater, plants, and foods). We cannot forget that free DNA can be taken up and able to transform other microorganisms to express novel, unexpected traits.

A wide range of applications is described by this collection of papers from wastewater, to plants, fruits, and foods. Again, each application presents different challenges and those needing to develop protocols for pathogen detection can use this Research Topic's collection to guide their investigations.

All of the detection protocols are described in detail along with the challenges identified by the individual investigations. As all the detection methodologies described in the Research Topic are novel and cutting-edge, they deserve publication and dissemination. I worry, however, that the level of technology development and cost may take them out of the market for application amongst regions of the world that lack the technological or economic development to employ these methods. We are all challenged to develop low-cost, rapid methods for pathogen-detection that can be used throughout our world.

## Author Contributions

All authors listed have made a substantial, direct, and intellectual contribution to the work and approved it for publication.

## Conflict of Interest

Y-WT was employed by Cepheid. The remaining authors declare that the research was conducted in the absence of any commercial or financial relationships that could be construed as a potential conflict of interest.

## Publisher's Note

All claims expressed in this article are solely those of the authors and do not necessarily represent those of their affiliated organizations, or those of the publisher, the editors and the reviewers. Any product that may be evaluated in this article, or claim that may be made by its manufacturer, is not guaranteed or endorsed by the publisher.

